# New depsidones and isoindolinones from the mangrove endophytic fungus *Meyerozyma guilliermondii* (HZ-Y_2_) isolated from the South China Sea

**DOI:** 10.3762/bjoc.11.133

**Published:** 2015-07-16

**Authors:** Senhua Chen, Zhaoming Liu, Yayue Liu, Yongjun Lu, Lei He, Zhigang She

**Affiliations:** 1 School of Chemistry and Chemical Engineering, Sun Yat-Sen University, 135 Xin gang West Road, Guangzhou 510275, China; 2 Guangdong Province Key Laboratory of Functional Molecules in Oceanic Microorganism, Bureau of Education, Sun Yat-Sen University, 74 Zhongshan Road II, Guangzhou 510080, China; 3 School of Life Sciences and Biomedical Center, Sun Yat-Sen University, 135 Xin gang West Road, Guangzhou 510275, China

**Keywords:** endophytic fungus, depsidone, α-glucosidase inhibitory activity, isoindolinone, *Meyerozyma guilliermondii*

## Abstract

Three new depsidones, botryorhodines E–G (**1**–**3**), and two new isoindolinones, meyeroguillines A and B (**7** and **9**), along with five known compounds were isolated from an endophytic fungus *Meyerozyma guilliermondii*, derived from the mangrove plant *Kandelia obovata.* Their structures were elucidated by 1D and 2D NMR spectroscopy and high resolution mass spectrometry (HREIMS). Compounds **1**–**6** exhibited strong α-glucosidase inhibitory activity with IC_50_ values ranging from 2.1 to 13.3 μM. Moreover, kinetic studies of compounds **2** and **6** showed that both of them were noncompetitive inhibitors of α-glucosidase.

## Introduction

Depsidones are characterized by the presence of cyclic diaryl ethers with an ester link joining the two aromatic rings [1–2]. They have been isolated mainly from various lichens [3–5] and other fungi [6–11]. These metabolites displayed a wide range of bioactivities including antibacterial [10], antifungal [6], antiviral [12], antioxidant [5], and cytotoxic [9].

**Figure 1:**
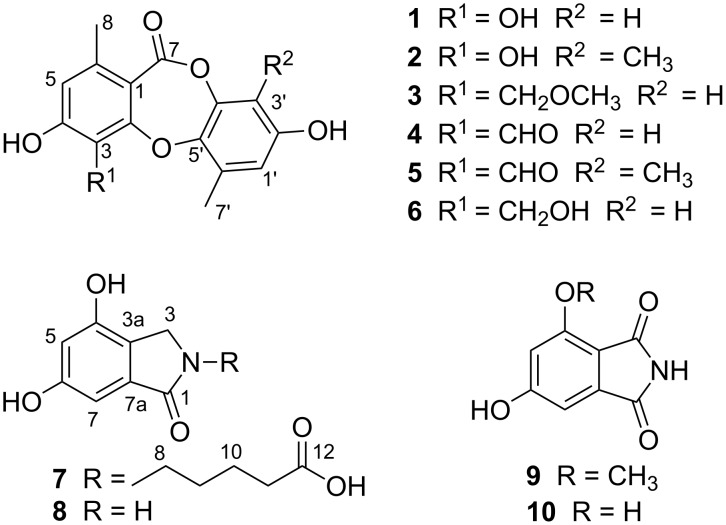
Chemical structures of **1**–**10**.

In the last decade, our research group has been focused on isolating novel compounds from endophytic fungi growing on mangroves in the South China Sea and investigating their bioactivity [13–17]. Recently, a chemical investigation of the endophytic fungal strain *Meyerozyma guilliermondii* (HZ-Y_2_), obtained from the roots of the mangrove plant *Kandelia obovata*, was carried out. The EtOAc extract of a fermentation broth of the fungus showed α-glucosidase inhibitory activity. Bioassay-guided fractionation of the bioactive extract led to the isolation of three new depsidones belonging to the analogues of the botryorhodines A–D [6], botryorhodines E–G (**1**–**3**), and two new isoindolinones, meyeroguillines A and B (**7** and **9**), together with five known compounds (**4**–**6**, **8** and **10**) (Figure 1). In the bioactivity assay, all depsidones showed strong *α*-glucosidase inhibitory activity with IC_50_ values ranging from 2.1 to 13.3 μM. Details of the isolation, structure elucidation, and biological activity of these compounds are reported herein.

## Results and Discussion

**Figure 2:**
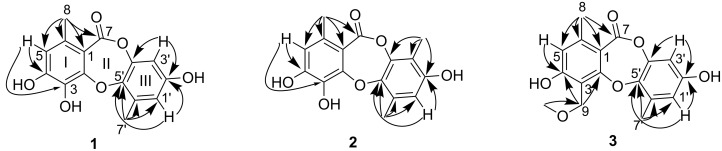
Selected HMBC (arrow) correlations of **1**–**3**.

Botryorhodine E (**1**) was obtained as a white, amorphous powder. The molecular formula was assigned to be C_15_H_12_O_6_, with ten degrees of unsaturation, based on its HREIMS (*m*/*z* 288.0627, calcd for C_15_H_12_O_6_, 288.0628). The ^1^H NMR spectrum exhibited three aromatic protons (δ_H_ 6.55, 6.45, 6.42), and two aromatic methyl groups (δ_H_ 2.31, 2.47). The ^13^C NMR and DEPT spectra revealed the presence of 15 carbons (Table 1), of which 12 aromatic carbons for two phenyl units, one quaternary carbon belonging to a carbonyl carbon, and the other two sp^3^ carbons corresponding to two methyls. The HMBC correlations from H-5 to C-8, C-7, C-4, C-3, C-1 and H-8 to C-7, C-6, C-5, C-1 indicated the presence of a 1,2-disubstitited 6-methyl-3,4-dihdroxyphenyl ring (I) (Figure 2). The second phenyl group was substituted with two *meta* coupled protons at δ_H_ 6.45 (1H, d, *J* = 2.8, H-3′) and δ_H_ 6.42 (1H, d, *J* = 2.8, H-1′) as well as a methyl group at δ_H_ 2.47 (H-7′) whose protons correlated to C-6′, C-5′ and C-1′ in the HMBC. The HMBC correlations from H-3′ to C-5′, C-4′ and H-1′ to C-5′ further established the second aromatic ring (III). The remaining carbon at δ_C_ 165.9 (C-7) indicated a carbonyl ester as a result of the observation of a strong absorption at *ν*_max_ 1680 cm^−1^ in the IR spectrum. It could be adjacent to C-1 due to contrary to the downfield shift observed for the oxygen bonded carbons C-2 (δ_C_ 152.4), C-5′ (δ_C_ 144.2), C-4′ (δ_C_ 146.3) and an upfield shift observed for C-1 (δ_C_ 113.7). The required degrees of unsaturation suggested that the aromatic rings (I) and (III) should be linked by ether and an ester bridge, revealing a depsidone skeleton with a seven-membered ring (II). Thus, the structure of **1** was revealed as a new depsidone belonging to the same family compound of the botryorhodines A–D [6], named botryorhodine E.

**Table 1:** ^1^H and ^13^C NMR spectroscopic data for compounds **1**–**3**.

Position	**1**^a^		**2**^a^		**3**^b^	

	δ_C_	δ_H_ (J in Hz)	δ_C_	δ_H_ (J in Hz)	δ_C_	δ_H_ (J in Hz)

1	113.7, qC		113.7, qC		114.0, qC	
2	152.4, qC		152.5, qC		162.3, qC	
3	135.0, qC		135.1, qC		116.3, qC	
4	152.4, qC		152.4, qC		161.6, qC	
5	115.7, CH	6.55, s	115.7, CH	6.54, s	116.3, CH	6.69, s
6	135.0, qC		135.0, qC		145.7, qC	
7	165.9, qC		166.1, qC		163.7, qC	
8	20.6, CH_3_	2.31, s	20.6, CH_3_	2.32, s	21.3, CH_3_	2.38, s
9					58.5, CH_2_	4.78, s
1′	114.9, CH	6.42, d (2.8)	113.8, CH	6.43, s	114.8, CH	6.53, s
2′	155.7, qC		153.7, qC		155.5, qC	
3′	105.7, CH	6.45, d (2.8)	115.0, qC		105.9, CH	6.53, s
4′	146.3, qC		144.9, qC		145.5, qC	
5′	144.2, qC		144.6, qC		143.2, qC	
6′	133.2, qC		128.9, qC		132.6, qC	
7′	16.6, CH_3_	2.47, s	16.3, CH_3_	2.43, s	16.7, CH_3_	2.43, s
8′			9.2, CH_3_	2.12, s		
9-OCH_3_					64.5, CH_3_	3.42, s

^a^Spectra were recorded at 500 MHz for ^1^H and 125 MHz for ^13^C in CD_3_OD. ^b^Spectra were recorded at 400 MHz for ^1^H and 100 MHz for ^13^C in acetone-*d*_6_.

Botryorhodine F (**2**), a minor product from the fraction of compound **1**, was obtained as an amorphous powder, deduced the molecular formula C_16_H_14_O_6_ from its HREIMS (*m*/*z* 302.0787, calcd for C_16_H_14_O_6_, 302.0785). The ^1^H and ^13^C NMR spectra of compound **2** were similar to those of compound **1**, except for the absence of an aromatic proton at δ_H_ 6.45 and the presence of a methyl group at δ_H_ 3.42 (δ_C_ 9.2). This suggested that compound **2** was a homologue of compound **1** with the replacement of an aromatic proton by a methyl group, which was supported by the HMBC correlations from H-8′ (δ_H_ 2.12) to C-4′, C-3′ and C-2′ (Figure 2). Detailed analysis of the 2D NMR spectroscopic data, the structure of **2** was established as a 3′-methylated analogue of **1**.

Botryorhodine G (**3**) was isolated as a white powder. Its molecular formula was determined as C_16_H_14_O_6_ by HRESIMS. The ^1^H and ^13^C NMR spectral data (Table 1) was greatly similar to those of compound **1** suggesting that both compounds have the same basic framework. The main difference between the two compounds was a hydroxy group at δ_C_ 135.0 (C-3) in **1** replaced by a methoxymethyl group in **3**, which was supported by HMBC correlations of 9-OCH_3_ to C-9 and H-9 to C-4, C-3, C-1. Therefore, the structure of compound **3** was elucidated as shown.

**Figure 3:**
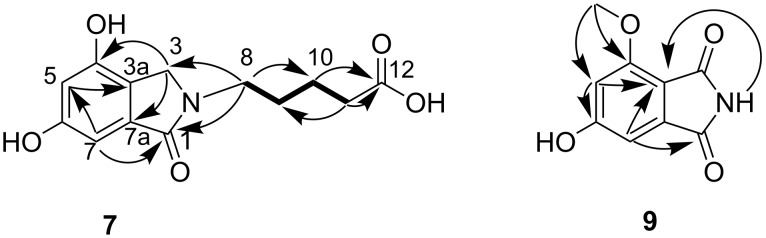
^1^H,^1^H COSY (bold) and selected HMBC (arrow) correlations of **7** and **9**.

Meyeroguilline A (**7**) was obtained as an amorphous powder. The molecular formula was established by analysis of the HREIMS (*m*/*z* 265.0946 calcd for C_13_H_15_O_5_N, 265.0945) in combination with ^1^H and ^13^C NMR data, indicating seven degrees of unsaturation. The UV spectral data at 242 (4.30), 291 (4.02), and 326 (3.86) nm indicated the existence of a benzoyl group. The ^1^H NMR spectrum (Table 2) along with HSQC spectrum showed signals due to the presence of five methylene protons (δ_H_ 1.44−4.19), two phenolic hydroxy groups (δ_H_ 9.89 and 9.55), and two aromatic protons (δ_H_ 6.43 and 6.48), showing a typical pattern of meta-coupling (*J* = 1.8 Hz) consistent with a 1,2,3,5-tetrasubstituted benzene moiety. Moreover, two carbonyl groups (δ_C_ 167.8 and 174.4) were clearly seen in the ^13^C NMR spectrum. Taking into account the required degrees of unsaturation, compound **7** contained a bicyclic aromatic lactam fragment. Further detailed analysis of the ^1^H and ^13^C NMR spectra suggested that **7** is an isoindolinone derivative [18]. Analysis of the ^1^H,^1^H COSY spectrum suggested the presence of one spin system, which included H-8/H-9/H-10/H-11 (Figure 3). In the HMBC spectrum (Figure 3), the correlations of H-10 and H-11 to C-12 (carbonyl), H-8 to C-10, and H-11 to C-9, established a valeric acid moiety. The linkage of valeric acid moiety to N-2 of the isoindolinone was assigned by HMBC correlations from H-8 to C-1 and C-3. The two aromatic hydroxy groups were accommodated at C-4 and C-6, based on the HMBC corrections from H-3 to C-4 (δ_C_ 153.2) and H-7 to C-6 (δ_C_ 158.8), respectively. To the best of our knowledge, compound **7** possessed a valeric acid moiety was the first reported example of a natural isoindolinone.

**Table 2:** ^1^H and ^13^C NMR spectroscopic data for compounds **7** and **9** in DMSO-*d*_6_.

Position	**7**^a^		**9**^a^	

	δ_C_	δ_H_ (*J* in Hz)	δ_C_	δ_H_ (*J* in Hz)

1	167.8, qC		169.2, qC	
3	46.9, CH_2_	4.19, s	167.7, qC	
3a	118.4, qC		109.2, qC	
4	153.2, qC		158.5, qC	
5	105.4, CH	6.43, d (1.75)	104.3, CH	6.68, br s
6	158.8, qC		165.6, qC	
7	100.1, CH	6.48, d (1.75)	102.8, CH	6.70, br s
7a	134.9, qC		137.3, qC	
8	41.2, CH_2_	3.46, t (6.74)		
9	27.2, CH_2_	1.58, m		
10	21.8, CH_2_	1.44, m		
11	33.2, CH_2_	2.24, t (7.25)		
12	174.4, qC			
4-OCH_3_			56.3	3.86
2-NH				10.83

^a^Spectra were recorded at 400 MHz for ^1^H and 100 MHz for ^13^C.

Meyeroguilline B (**9**) was isolated as a pale yellow amorphous powder, the molecular formula was assigned as C_9_H_7_O_4_N based on HREIMS (*m*/*z* 193.0370). Nine signals in the ^13^C NMR were classified by the DEPT spectra, including a methyl, two methine, and six quaternary carbons (Table 2). The ^1^H NMR and HSQC spectra revealed a methoxy group (δ_H_ 3.86), two benzene protons (δ_H_ 6.68 and 6.70). The NMR signals of **9** were principally similar to those of **10** [18], except that the chelated hydroxy group (4-OH in **10**) was replaced by a methoxy group (Figure 3). The HMBC correlations from 4-OCH_3_ to C-4 also indicated that the attachment of the methoxy group to C-4. The structure of **9** was thus established as 6-hydroxy-4-methoxyisoindoline-1,3-dione.

In addition, the structures of the known compounds were identified as botryorhodine A (**4**) [6], botryorhodine B (**5**) [6], botryorhodine D (**6**) [6], 4,6-dihydroxy-2,3-dihydro-1*H*-isoindol-1-one (**8**) [18], 4,6-dihydroxy-1*H*-isoindole-1,3(2*H*)-dione (**10**) [18], by comparison of their spectroscopic data with those reported in the literature.

**Figure 4:**
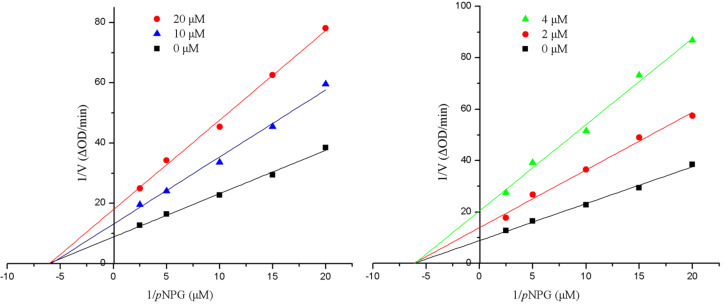
Kinetic analysis of the inhibition of α-glucosidase by **2** (left) and **6** (right).

The isolated compounds were tested for *α*-glucosidase inhibitory activity using acarbose as positive control. All depsidones **1**–**6** exhibited strong *α*-glucosidase inhibitory activity with IC_50_ (μM) values ranging 2.1–15.4 (Table 3). Compound **6** showed the best inhibitory activity (IC_50_ 2.1 μM) among the depsidones. Comparison with those depsidones, the structural differences mainly occur in the functional group of C-3 and C-3′, which indicated that the hydroxymethyl group at C-3 could enhance the α-glucosidase inhibitory activity, while the methyl group at C-3′ did not affect that activity. Moreover, kinetic studies of compounds **2** and **6** showed that both of them were noncompetitive inhibitors of α-glucosidase (Figure 4). Compound **7** showed the promising α-glucosidase inhibitory activity, whereas compound **8** and **10** inhibited α-glucosidase with moderate to weak activities (Table 3).

**Table 3:** α-Glucosidase inhibitory activities^a^.

Compounds	IC_50_ (μM)

**1**	15.4 ± 0.4
**2**	9.8 ± 0.3
**3**	12.4 ± 0.4
**4**	13.3 ± 0.3
**5**	11.7 ± 0.4
**6**	2.1 ± 0.2
**7**	50.3 ± 1.3
**8**	180.1 ± 1.8
**10**	251.3 ± 2.5
Acarbose^b^	553.7 ± 2.4

^a^IC_50_ values are shown as mean ± SD from three independent experiments. The inhibitory activity of compound **9** were not tested due to the limited amount; ^b^positive control.

All isolated compounds were also evaluated for their inhibitory activity against Mycobacterium tuberculosis protein tyrosine phosphatase B (MptpB), of which were inactive (IC_50_ > 200 μM).

## Experimental

**General experimental procedures.** The NMR spectra were performed on Bruker Avance 400 spectrometer (^1^H 400 MHz, ^13^C 100 MHz) and Bruker Avance 500 spectrometer (^1^H 500 MHz, ^13^C 125 MHz). All chemical shifts (δ) are given in ppm with reference to the solvent signal (δ_C_ 49.0/δ_H_ 3.31 for CD_3_OD, δ_C_ 39.5/*δ*_H_ 2.50 for DMSO and δ_C_ 29.8/δ_H_ 2.05 for acetone-*d*_6_), and coupling constants (*J*) are given in Hz. UV data were measured on a PERSEE TU-1900 spectrophotometer. IR spectra were measured on a Nicolet Nexus 670 spectrophotometer, in KBr discs. EIMS on a DSQ EI-mass spectrometer (Thermo, Shanghai, China) and HREIMS data were measured on a DMAT95XP high-resolution mass spectrometer. ESIMS spectra were recorded on a Finnigan LCQ-DECA mass spectrometer and HRESIMS spectra were recorded on a Shimadzu LCMS-IT-TOF mass spectrometer. Column chromatography (CC) was performed on silica gel (200–300 mesh, Qingdao Marine Chemical Factory) and Sephadex LH-20 (Amersham Pharmacia, Piscataway). Precoated silica gel plates (Qingdao Huang Hai Chemical Group Co., G60, F-254) were used for thin-layer chromatography. Semipreparative HPLC was performed on a Waters Breeze HPLC system using a Phenomenex Luna (Phenomenex, Torrance) C_18_ column (250 × 10 mm, 5 μm).

**Fungal material.** The fungus used in this study was isolated from healthy roots of *Kandelia obovata*, which were collected in April 2012 from Huizhou Mangrove Nature Reserve in Guangdong Province, China. It was obtained using the standard protocol for the isolation. Fungal identification was carried out using a molecular biological protocol by DNA amplification and sequencing of the ITS region. The sequence data obtained from the fungal strain have been deposited at GenBank with accession no. KP975418. A BLAST search result showed that the sequence was the most similar (99%) to the sequence of *Meyerozyma guilliermondii* (compared to KF710038.1 FJ662408.1)*.* A voucher strain was deposited in School of Chemistry and Chemical Engineering, Sun Yat-Sen University, Guangzhou, China.

**Fermentation, extraction and isolation.** The fungus was grown on autoclaved rice solid-substrate medium (thirty 500 mL Erlenmeyer flasks, each containing 50 g rice and 50 mL distilled water) at room temperature under static conditions and daylight for 28 days. Following incubation, the mycelia and solid rice medium were extracted with EtOAc. The extract was evaporated under reduced pressure to yield 21 g of residue. The residue was then divided into 36 fractions (Fr. 1–Fr. 36) by column chromatography on silica gel eluted by a gradient of petroleum ether/EtOAc from 1:0 to 0:1. Fr. 6 (230 mg) was applied to silica gel CC, eluted with petroleum ether/EtOAc (3:1), to obtain compounds **4** (10 mg) and **5** (6 mg). Fr. 8 (134 mg) was subsequently separated by Sephadex LH-20 CC eluted with MeOH to give subfraction Fr. 11.8, which was purified on silica gel (petroleum ether/EtOAc v/v, 7:3) to yield **3** (3.7 mg) and **6** (2.5 mg). Fr. 15 was chromatographed on Sephadex LH-20 CC (CHCl_3_/MeOH v/v, 1:1) to give subfraction Fr. 15.5, which was puriﬁed using semipreparative reversed-phase HPLC (MeOH/H_2_O, 80:20; 2.0 mL/min) to yield **1** (3.3 mg, *t*_R_ = 18 min) and **2** (2.1 mg, *t*_R_ = 20 min). Fr. 20.11 was applied to Sephadex LH-20 column eluted with MeOH to obtain **10** (15 mg). Fr. 21 (304 mg) was rechromatographed on silica gel (gradient of CHCl_3_/MeOH from 1:0 to 1:1) to give subfraction Fr. 22.8, which was puriﬁed by Sephadex LH-20 CC (CHCl_3_/MeOH v/v, 1:1) to yield **8** (1.3 mg). Fr. 25 (80 mg) was subsequently separated by Sephadex LH-20 CC eluted with MeOH to obtain **7** (2.4 mg) and **9** (3.5 mg), respectively.

Botryorhodine E (**1**): white amorphous powder; UV (MeOH) λ_max_ (log ε): 262 (4.25), 364 (3.56) nm; IR (KBr) *ν*_max_: 3426, 2922, 2853, 1680, 1620, 1506, 1458, 1383, 1265, 1193, 1074, 908, 877, 809 cm^−1^; EIMS (*m*/*z*): 288; HREIMS (*m/z*): 288.0627 (calcd for C_15_H_12_O_6_, 288.0628); ^1^H NMR (CD_3_OD, 500 MHz) and ^13^C NMR (CD_3_OD, 125 MHz), see Table 1.

Botryorhodine F (**2**): white amorphous powder; UV (MeOH) λ_max_ (log ε): 262 (4.25), 364 (3.56) nm; IR (KBr) *ν*_max_: 3426, 2922, 2853, 1686, 1620, 1587, 1458, 1357, 1284, 1193, 1155, 1074, 915, 875, 812 cm^−1^; EIMS (*m*/*z*): 302; HREIMS (*m/z*): 302.0787 (calcd for C_16_H_14_O_6_, 302.0785); ^1^H NMR (CD_3_OD, 500 MHz) and ^13^C NMR (CD_3_OD, 125 MHz), see Table 1.

Botryorhodine G (**3**): white amorphous powder; UV (MeOH) λ_max_ (log ε): 262 (4.25), 364 (3.56) nm; IR (KBr) *ν*_max_: 3342, 3076, 2931, 2723, 1703, 1606, 1502, 1456, 1284, 1203, 1146, 1080, 995, 945, 850 cm^−1^; ESIMS (*m*/*z*): [M − H]^−^ 315; HRESIMS (*m*/*z*): [M − H]^−^ 315.0869 (calcd for C_17_H_15_O_6_, 315.0863); ^1^H NMR (acetone-*d*_6_, 400 MHz) and ^13^C NMR (acetone-*d*_6_, 100 MHz), see Table 1.

Meyeroguilline A (**7**): amorphous powder; UV (MeOH) λ_max_ (log ε): 242 (4.30), 291 (4.02), 326 (3.86) nm; IR (KBr) *ν*_max_: 3448, 2927, 2853, 1644, 1610, 1460, 1315, 1166, 1013, 816 cm^−1^; EIMS (*m*/*z*): 265; HREIMS (*m*/*z*): 265.0946 (calcd for C_13_H_15_O_5_N, 265.0945); ^1^H NMR (DMSO, 400 MHz) and ^13^C NMR (DMSO, 100 MHz), see Table 2.

Meyeroguilline B (**9**): amorphous powder; UV (MeOH) λ_max_ (log ε): 219 (4.25), 240 (3.94), 336 (3.56) nm; IR (KBr) *ν*_max_: 3438, 2926, 2853, 1648, 1609, 1459, 1264, 1165, 1015, 809 cm^−1^; EIMS (*m*/*z*): 193; HREIMS (*m*/*z*): 193.0370 (calcd for C_9_H_7_O_4_N, 193.0370); ^1^H NMR (DMSO, 400 MHz) and ^13^C NMR (DMSO, 100 MHz), see Table 2.

**Biological assays.** The assays for α-glucosidase [19] and MptpB inhibition inhibitory activities [13] were carried out as described previously.

## Supporting Information

File 1 ^1^H, ^13^C, ^1^H,^1^H COSY, HSQC and HMBC NMR spectra of the new compounds.
