# Supplementary material for: New depsidones and isoindolinones from the mangrove endophytic fungus Meyerozyma guilliermondii (HZ-Y2) isolated from the South China Sea
Source: Beilstein J Org Chem. 2015 Jul 16;11:1187–93. doi: 10.3762/bjoc.11.133 (PMC4578337; doi:10.3762/bjoc.11.133)

Supporting Information

for

New depsidones and isoindolinones from the mangrove endophytic fungus *Meyerozyma guilliermondii* (HZ-Y2) isolated from the South China Sea

Senhua Chen[1,2], Zhaoming Liu[1,2], Yayue Liu[1,2], Yongjun Lu[2,3], Lei He[2]
and Zhigang She *[1,2]

Address: [1] School of Chemistry and Chemical Engineering, Sun Yat-Sen University, 135 Xin gang West Road, Guangzhou 510275, China, [2]Guangdong Province Key Laboratory of Functional Molecules in Oceanic Microorganism, Bureau of Education, Sun Yat-Sen University, 74 Zhongshan Road II, Guangzhou 510080, China and [3]School of Life Sciences and Biomedical Center, Sun Yat-Sen University, 135 Xin gang West Road, Guangzhou 510275, China

Email: Zhigang She - cesshzhg@mail.sysu.edu.cn
*Corresponding author

Figure S1 ^{1}H NMR spectrum of **1** in CD_3OD 3
Figure S2 ^{13}C NMR spectrum of **1** in CD_3OD 3
Figure S3 HSQC spectrum of **1** in CD_3OD 4
Figure S4 ^{1}H–^{1}H COSY spectrum of **1** in CD_3OD 4
Figure S5 HMBC spectrum of **1** in CD_3OD 5
Figure S6 ^{1}H NMR spectrum of **2** in CD_3OD 5
Figure S7 ^{13}C NMR spectrum of **2** in CD_3OD 6
Figure S8 HSQC spectrum of **2** in CD_3OD 6
Figure S9 ^{1}H–^{1}H COSY spectrum of **2** in CD_3OD 7
Figure S10 HMBC spectrum of **2** in CD_3OD 7
Figure S11 ^{1}H NMR spectrum of **3** in acetone-d_6 8
Figure S12 ^{13}C NMR spectrum of **3** in acetone-d_6 8
Figure S13 HSQC spectrum of **3** in acetone-d_6 9
Figure S14 ^{1}H–^{1}H COSY spectrum of **3** in acetone-d_6 9
Figure S15 HMBC spectrum of **3** in acetone-d_6 10
Figure S16 ^{1}H NMR spectrum of **7** in DMSO 10
Figure S17 ^{13}C NMR spectrum of **7** in DMSO 11
Figure S18 HSQC spectrum of **7** in DMSO 11
Figure S19 ^{1}H–^{1}H COSY spectrum of **7** in DMSO 12
Figure S20 HMBC spectrum of **7** in DMSO 12
Figure S21 ^{1}H NMR spectrum of **9** in DMSO 13
Figure S22 ^{13}C NMR spectrum of **9** in DMSO 13
Figure S23 HSQC spectrum of **9** in DMSO 14
Figure S24 HMBC spectrum of **9** in DMSO 14

Figure S1 ^{1}H NMR spectrum of **1** in CD_3OD

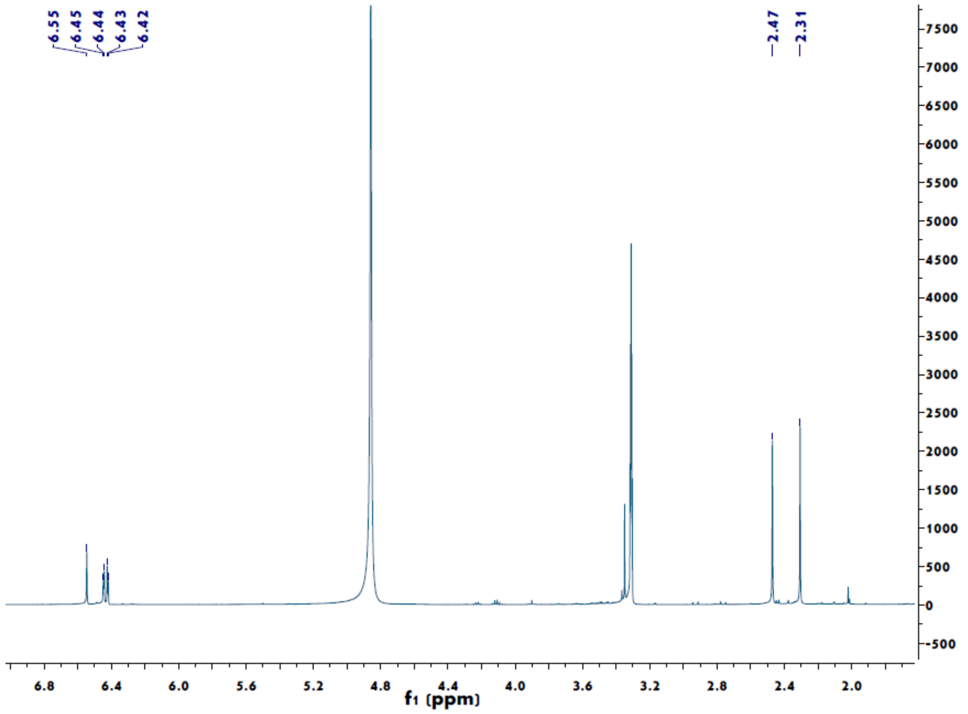

Figure S2 ^{13}C NMR spectrum of **1** in CD_3OD

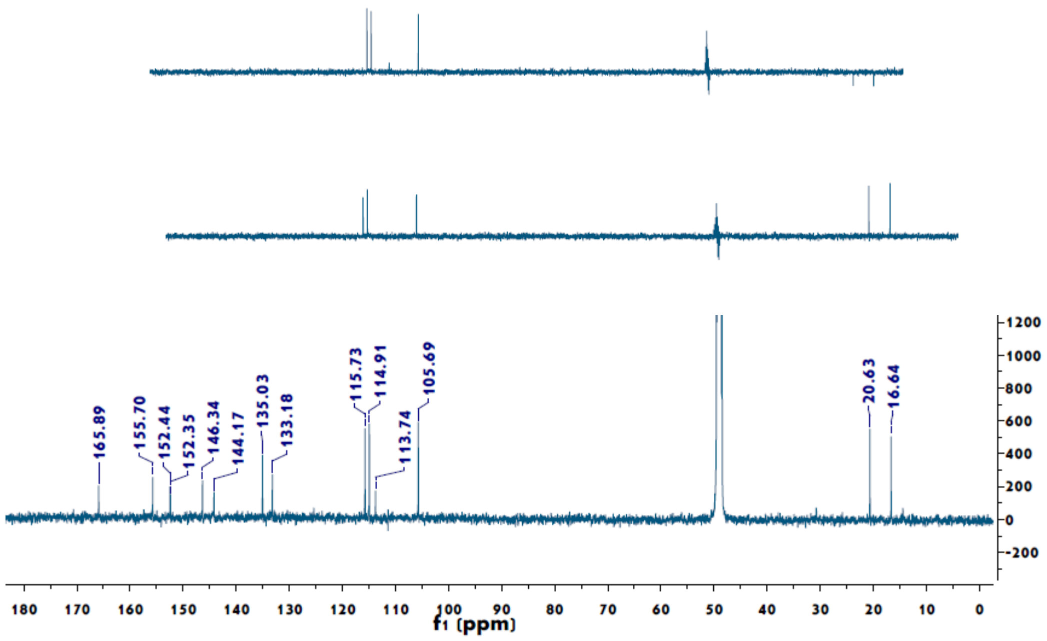

Figure S3 HSQC spectrum of **1** in CD_3OD

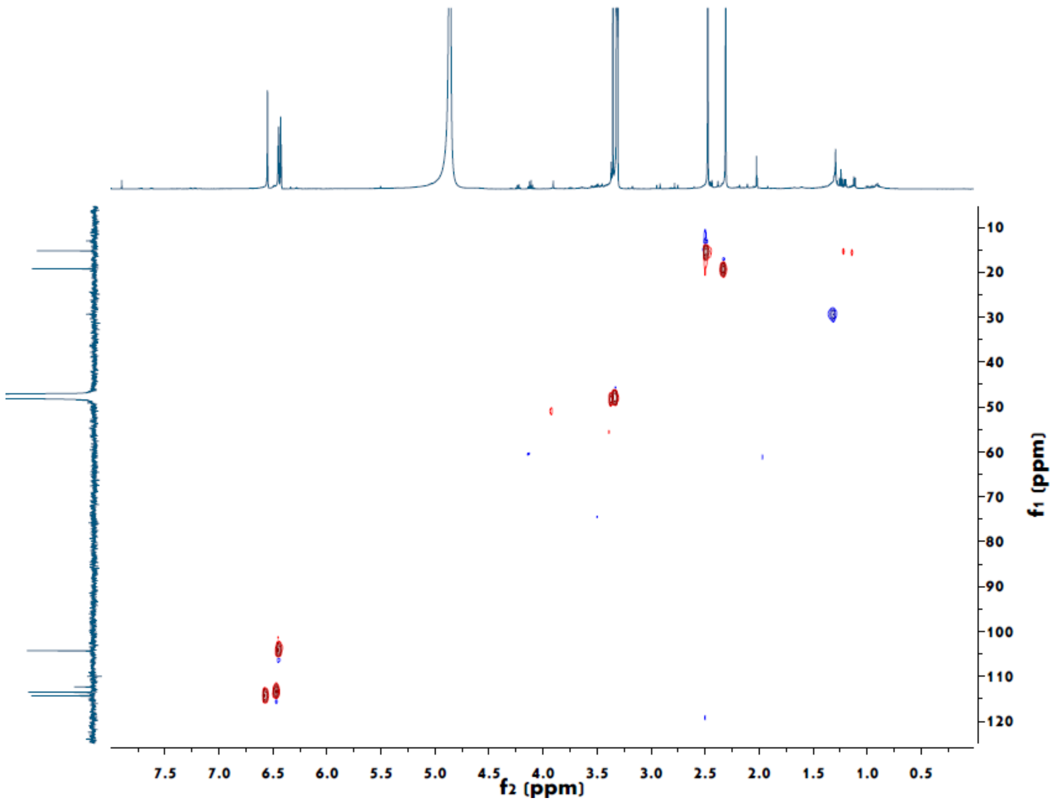

Figure S4 ^1H–^1H COSY spectrum of **1** in CD_3OD

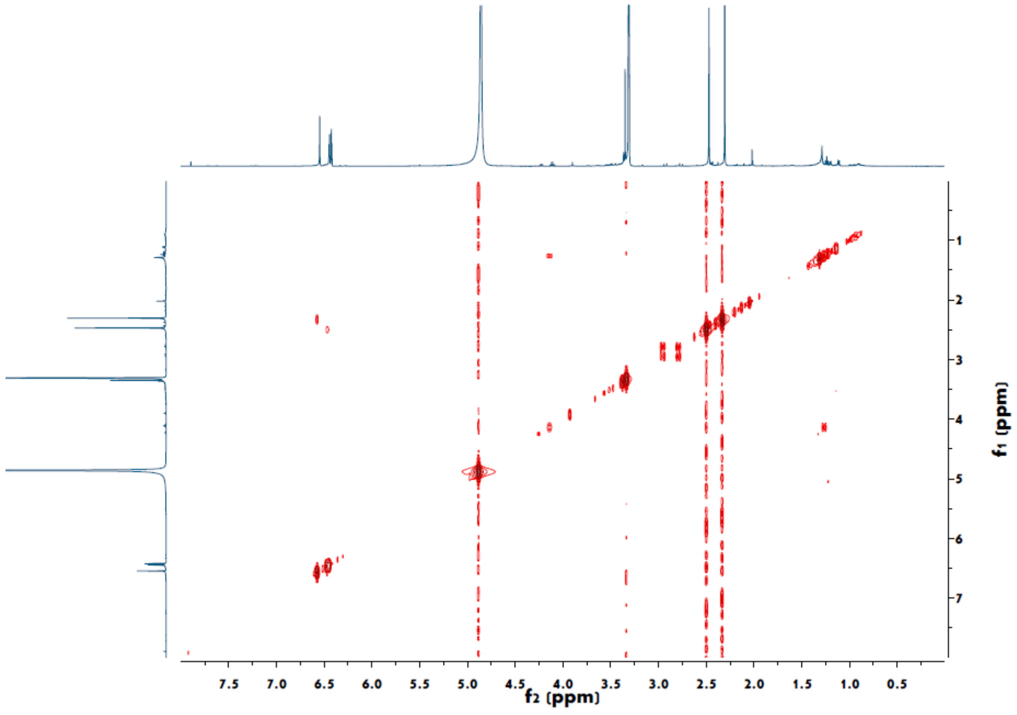

Figure S5 HMBC spectrum of **1** in CD_3OD

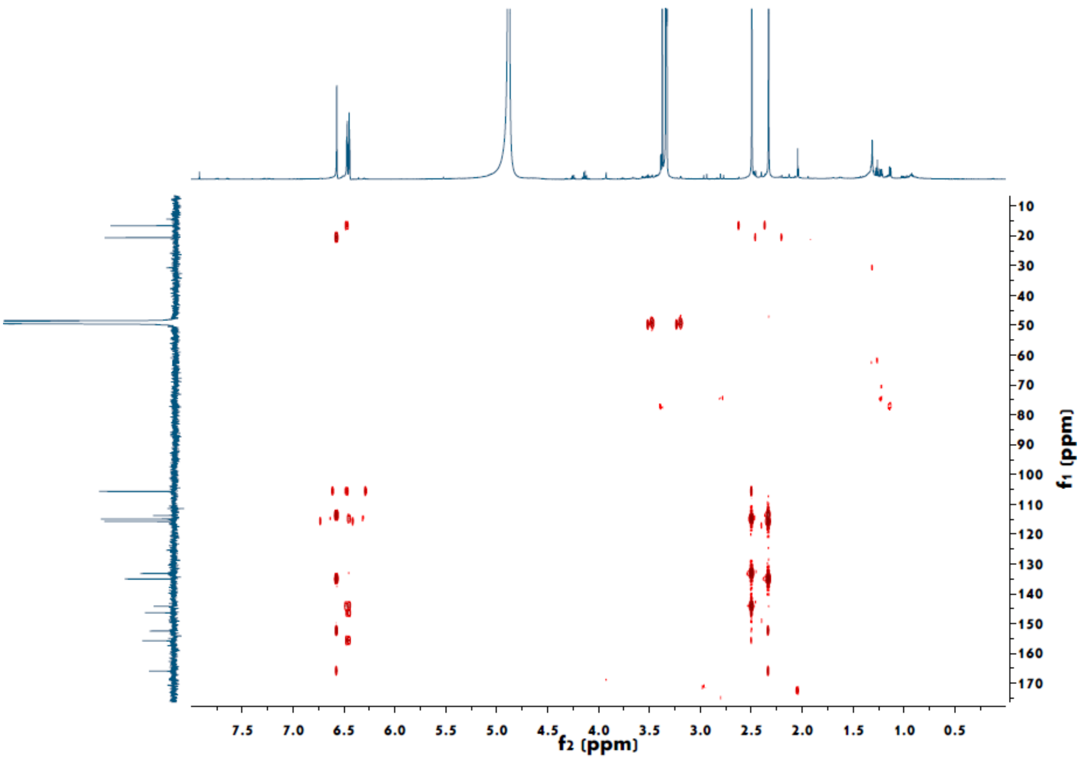

Figure S6 ^{1}H NMR spectrum of **2** in CD_3OD

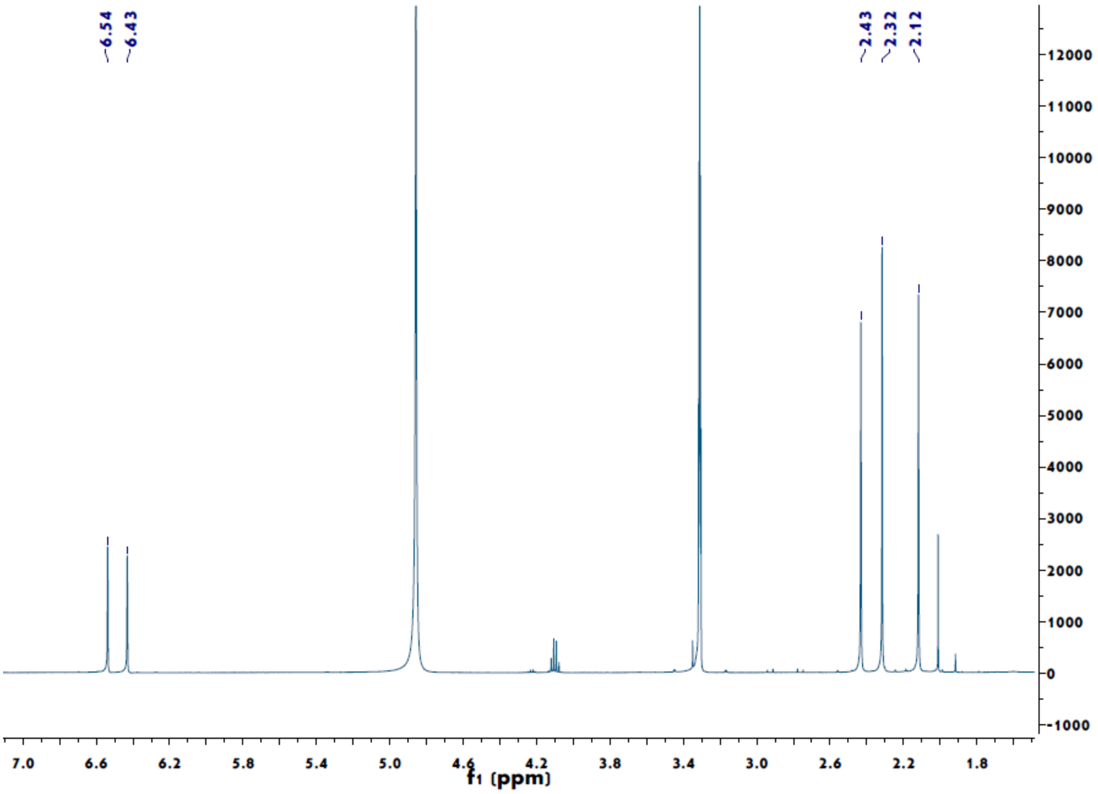

Figure S7 ^{13}C NMR spectrum of **2** in CD_3OD

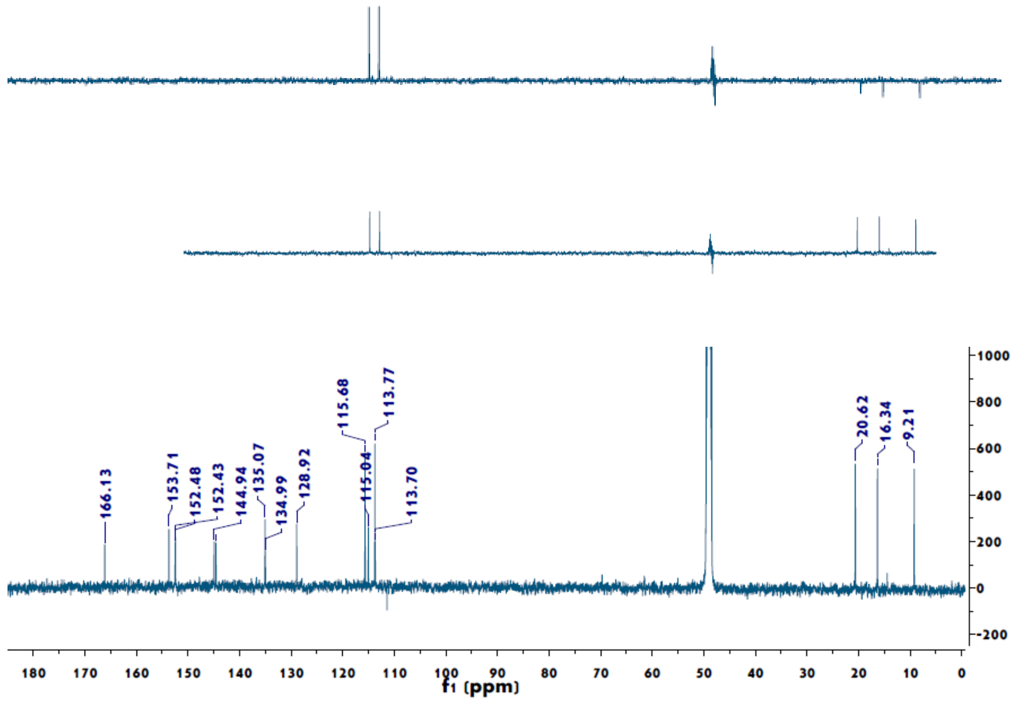

Figure S8 HSQC spectrum of **2** in CD_3OD

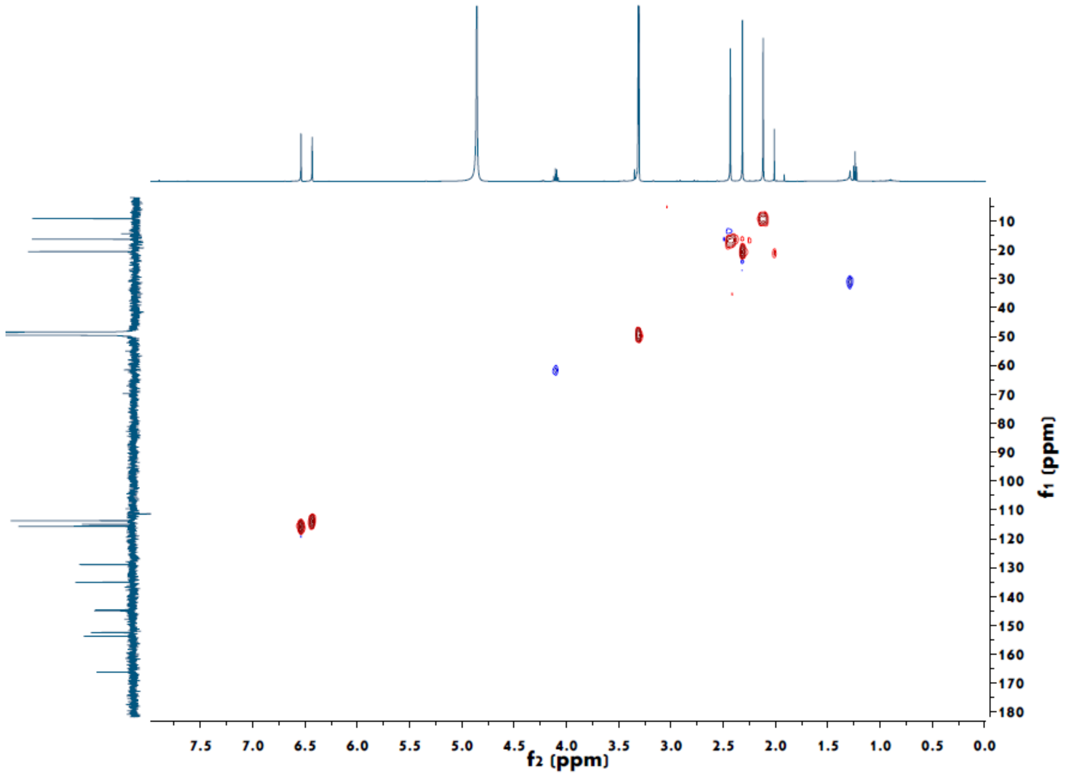

Figure S9 ^{1}H–^{1}H COSY spectrum of **2** in CD_3OD

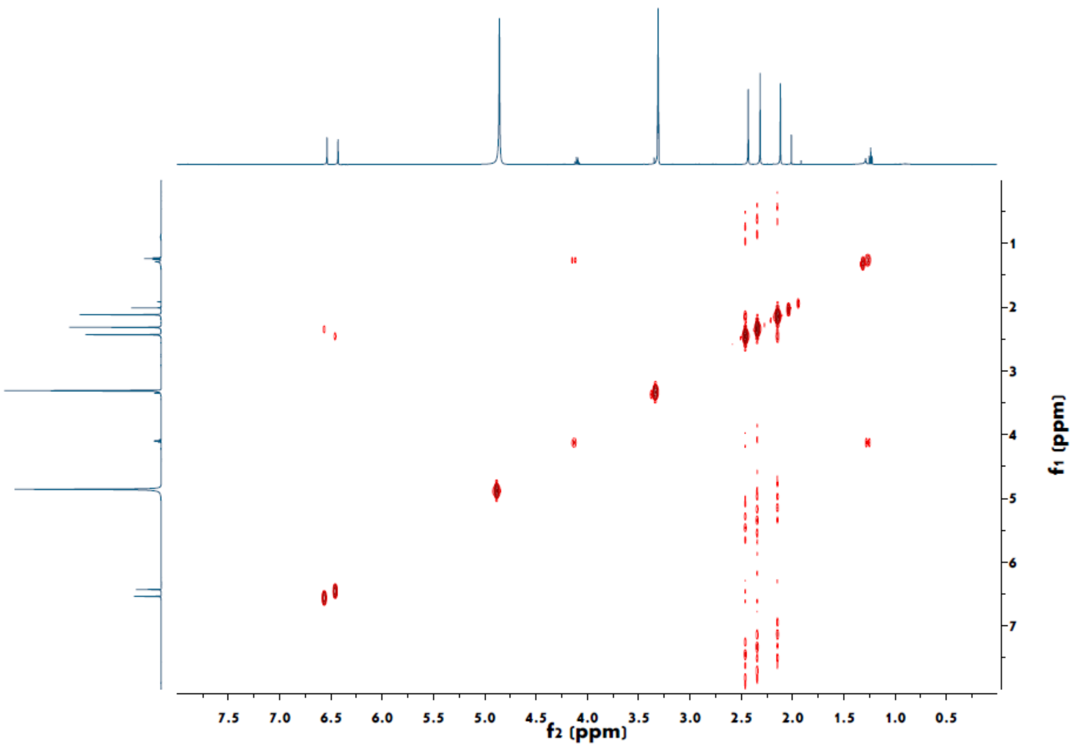

Figure S10 HMBC spectrum of **2** in CD_3OD

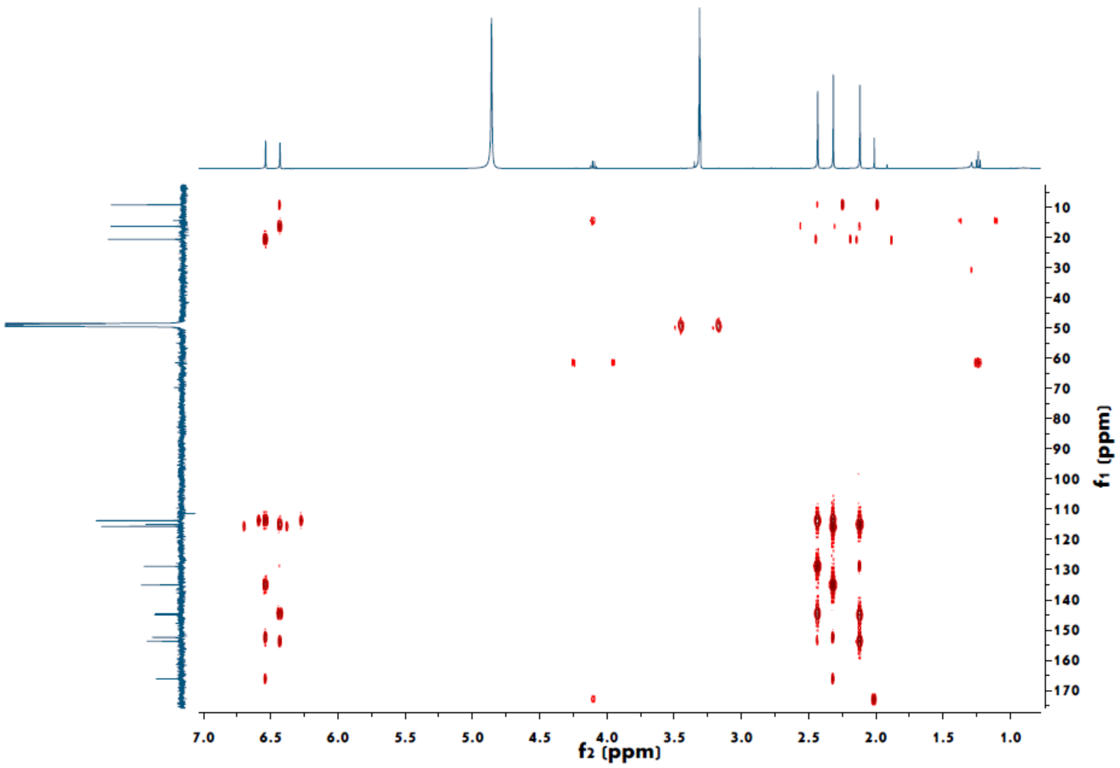

Figure S11 ^{1}H NMR spectrum of **3** in acetone-d_6

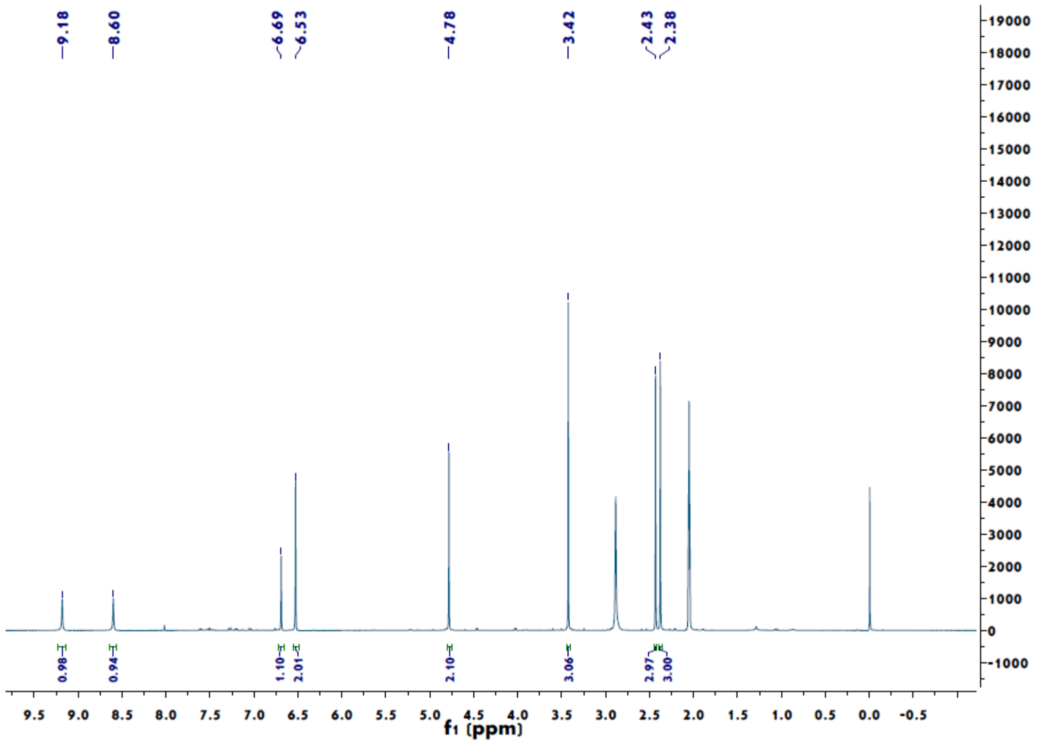

Figure S12 ^{13}C NMR spectrum of **3** in acetone-d_6

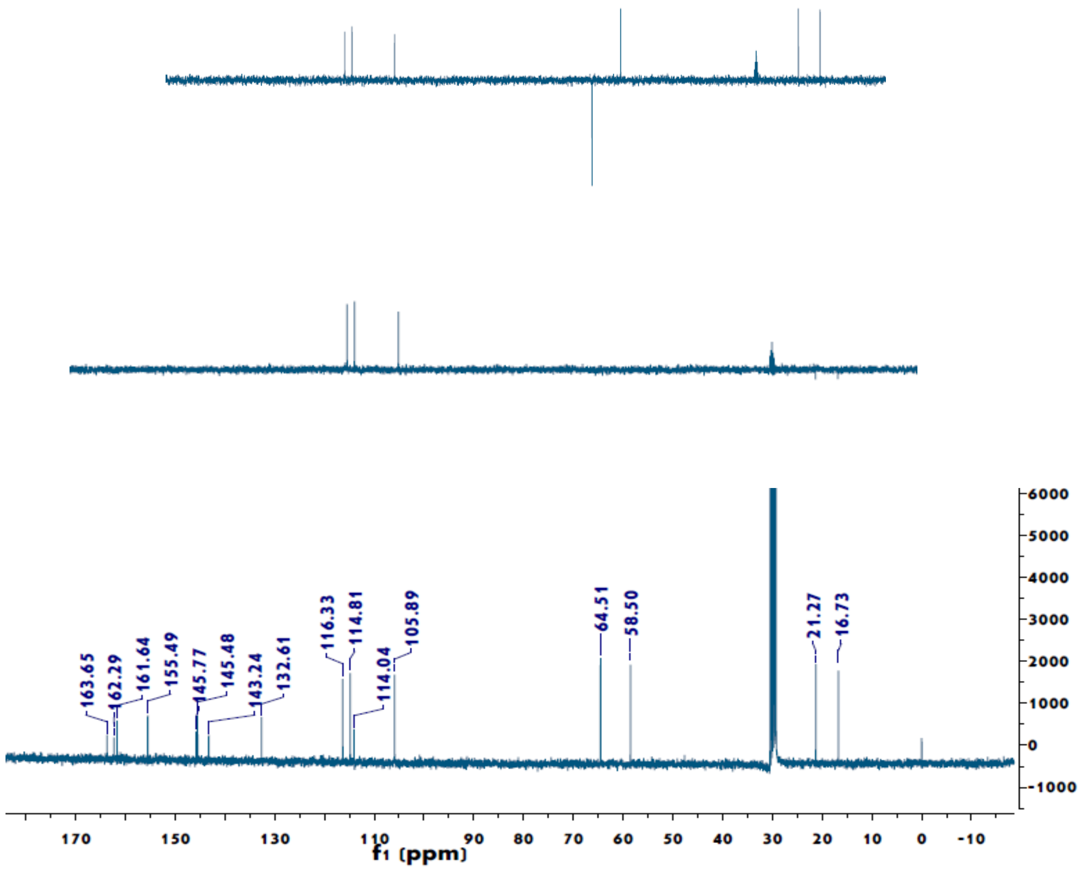

Figure S13 HSQC spectrum of **3** in acetone-d_6

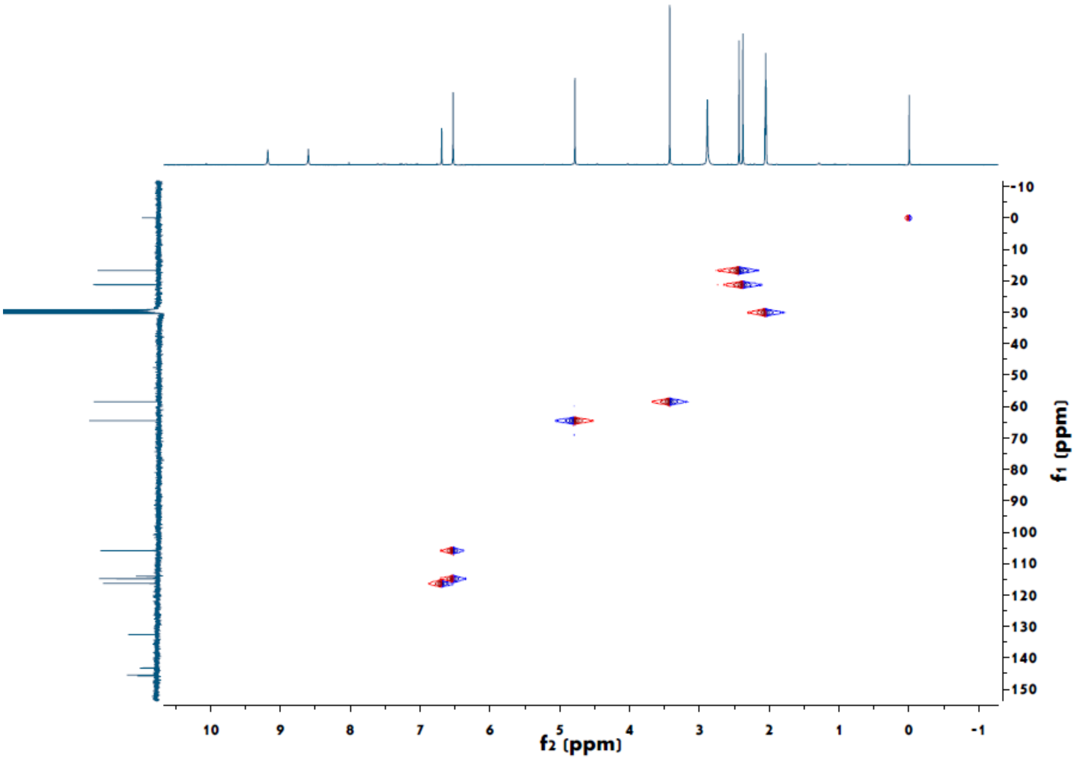

Figure S14 ^1H–^1H COSY spectrum of **3** in acetone-d_6

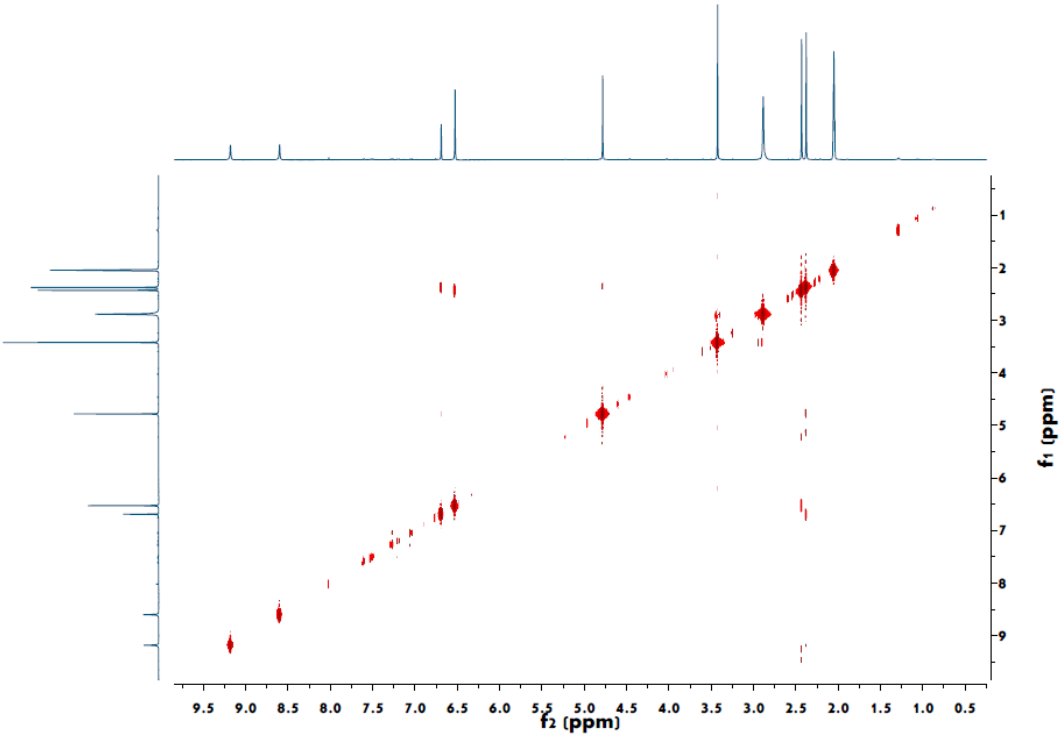

Figure S15 HMBC spectrum of **3** in acetone-d_6

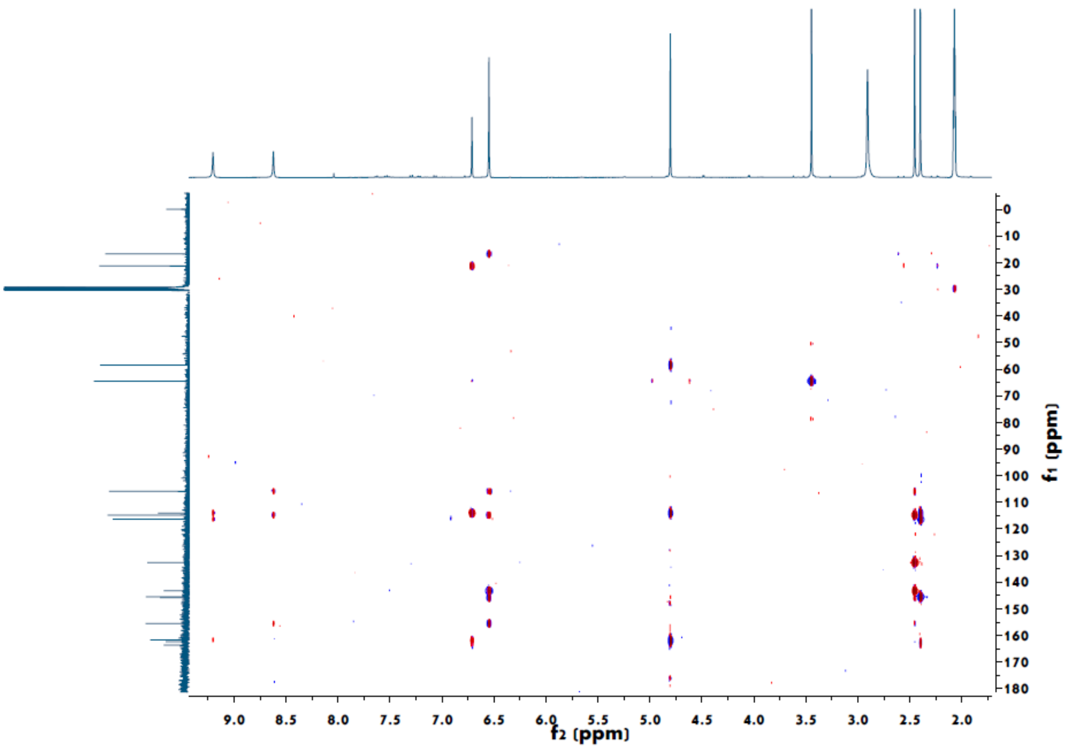

Figure S16 ^{1}H NMR spectrum of **7** in DMSO

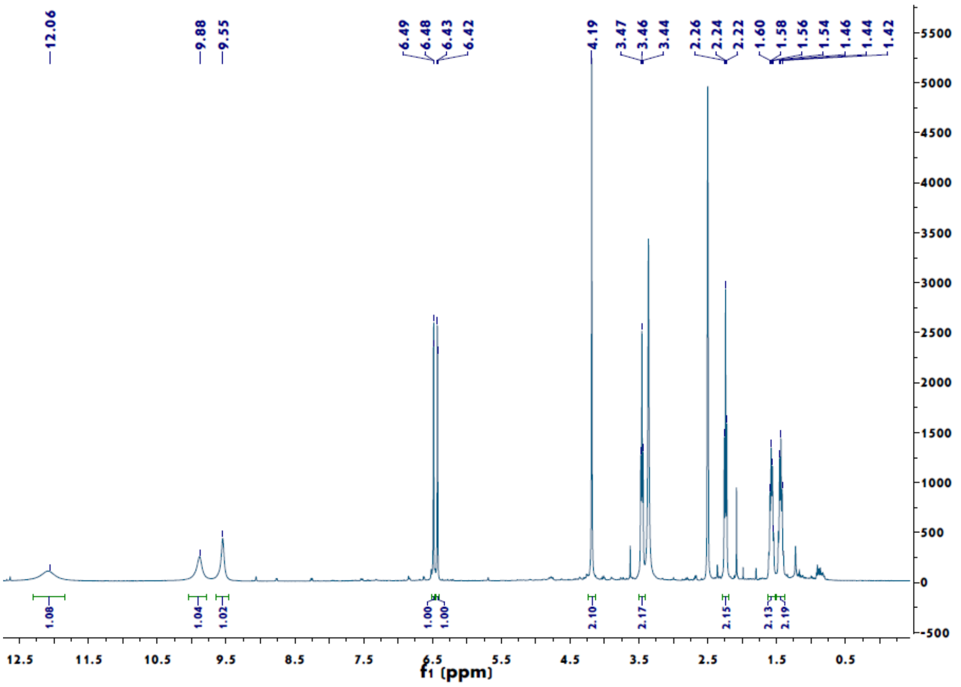

Figure S17 ^{13}C NMR spectrum of **7** in DMSO

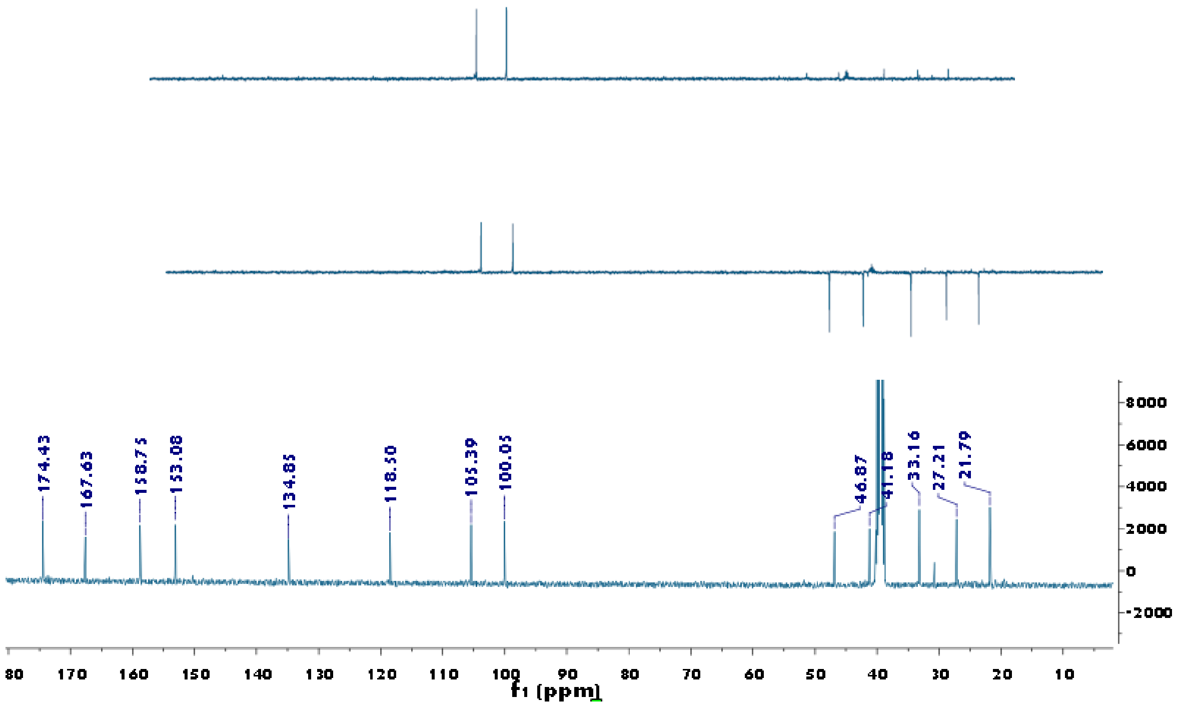

Figure S18 HSQC spectrum of **7** in DMSO

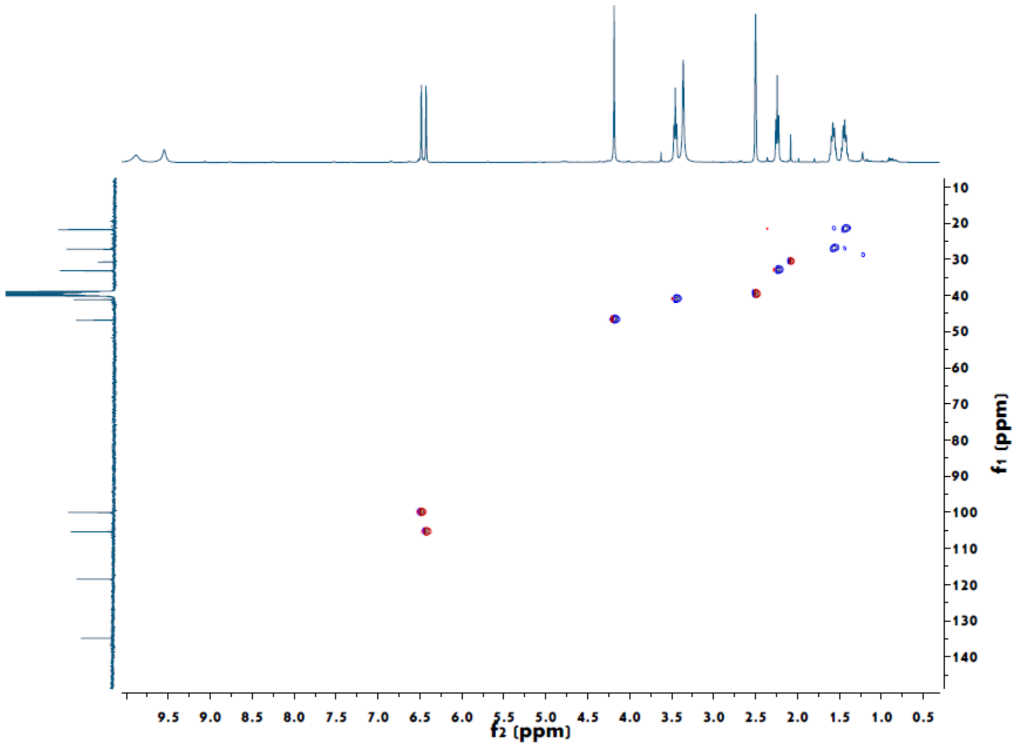

Figure S19 ^{1}H–^{1}H COSY spectrum of **7** in DMSO

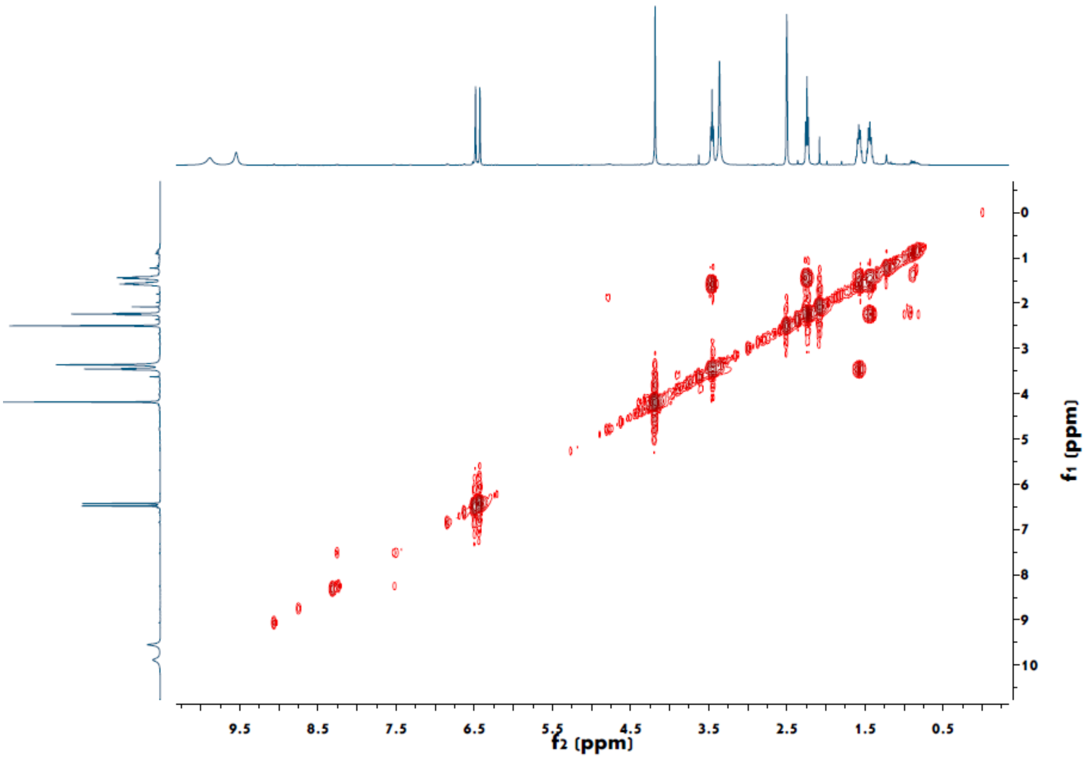

Figure S20 HMBC spectrum of **7** in DMSO

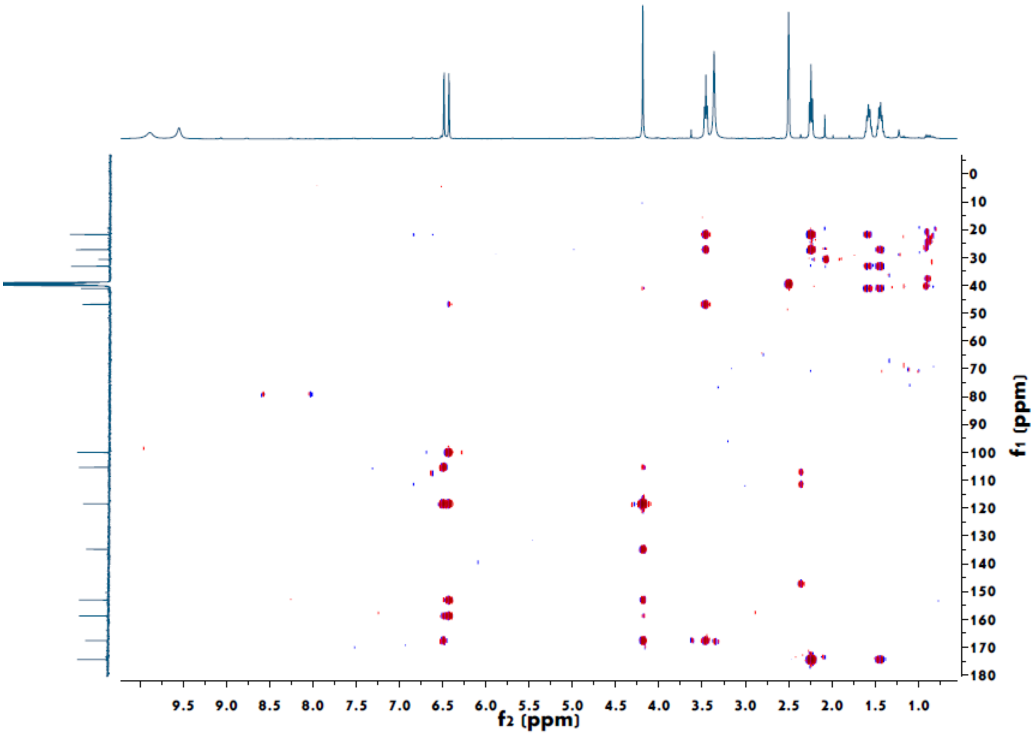

Figure S21 ^{1}H NMR spectrum of **9** in DMSO

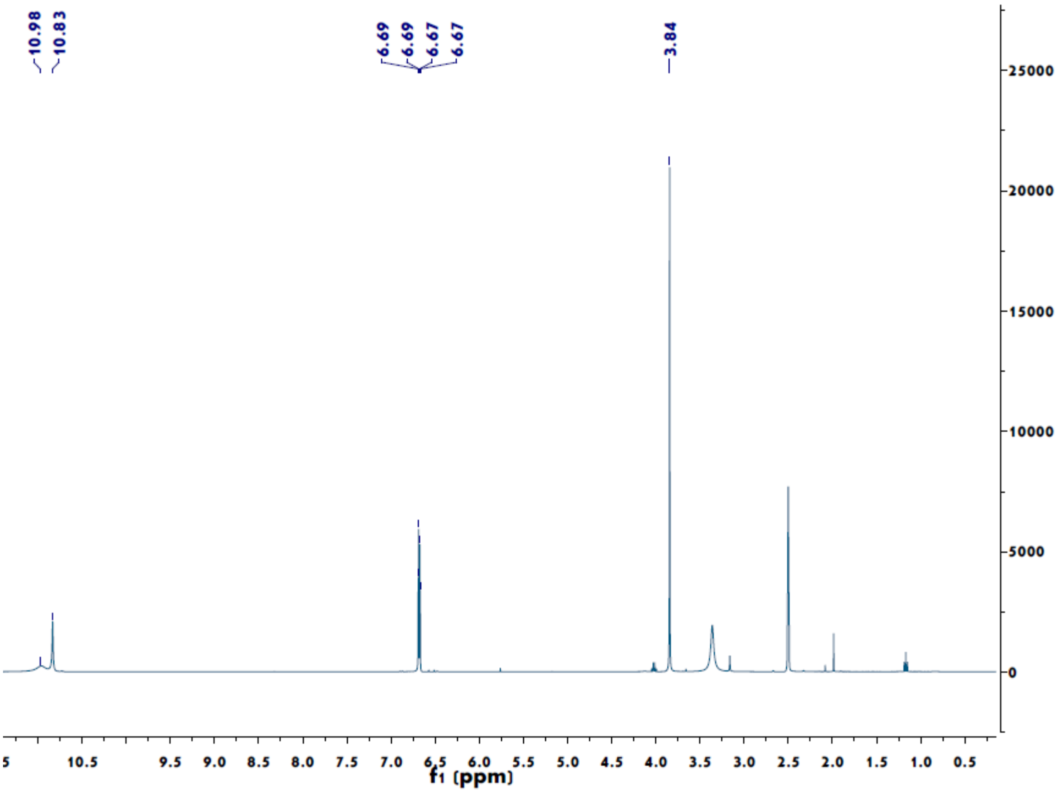

Figure S22 ^{13}C NMR spectrum of **9** in DMSO

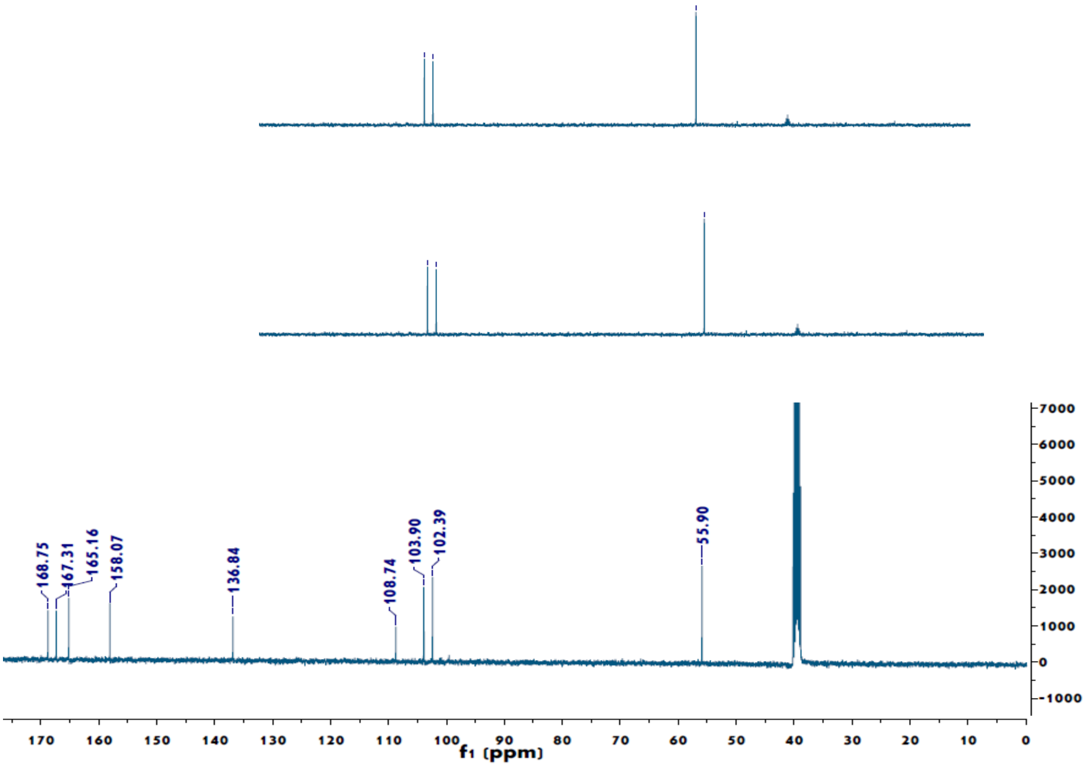

Figure S23 HSQC spectrum of **9** in DMSO

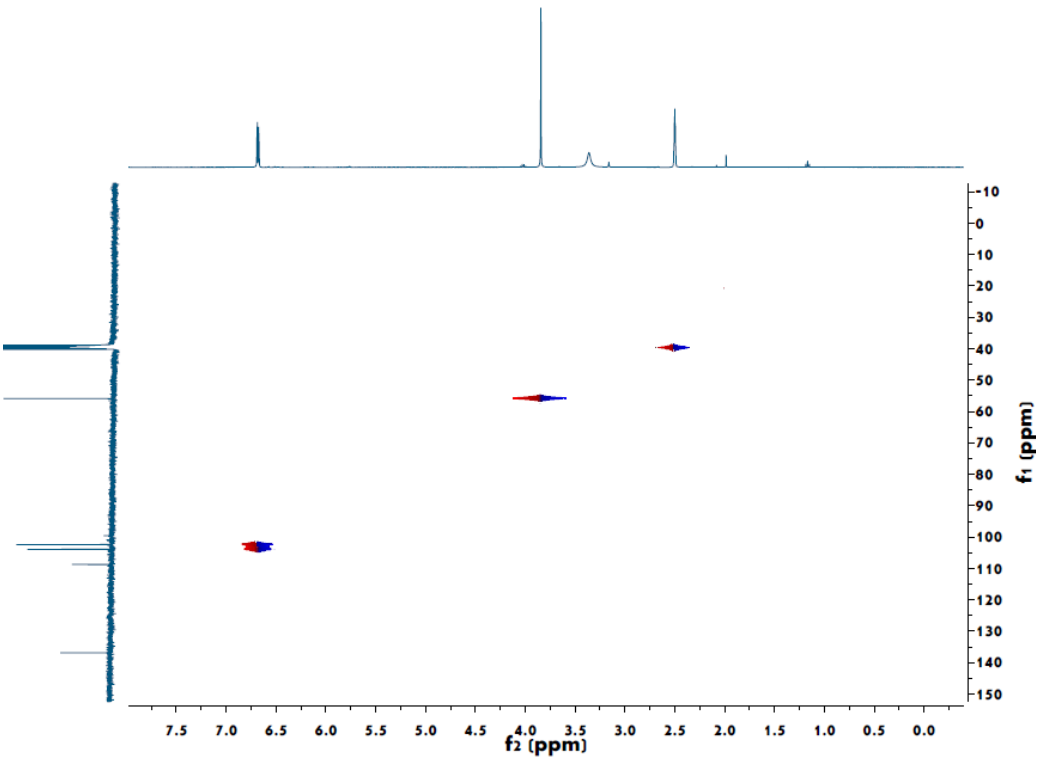

Figure S24 HMBC spectrum of **9** in DMSO

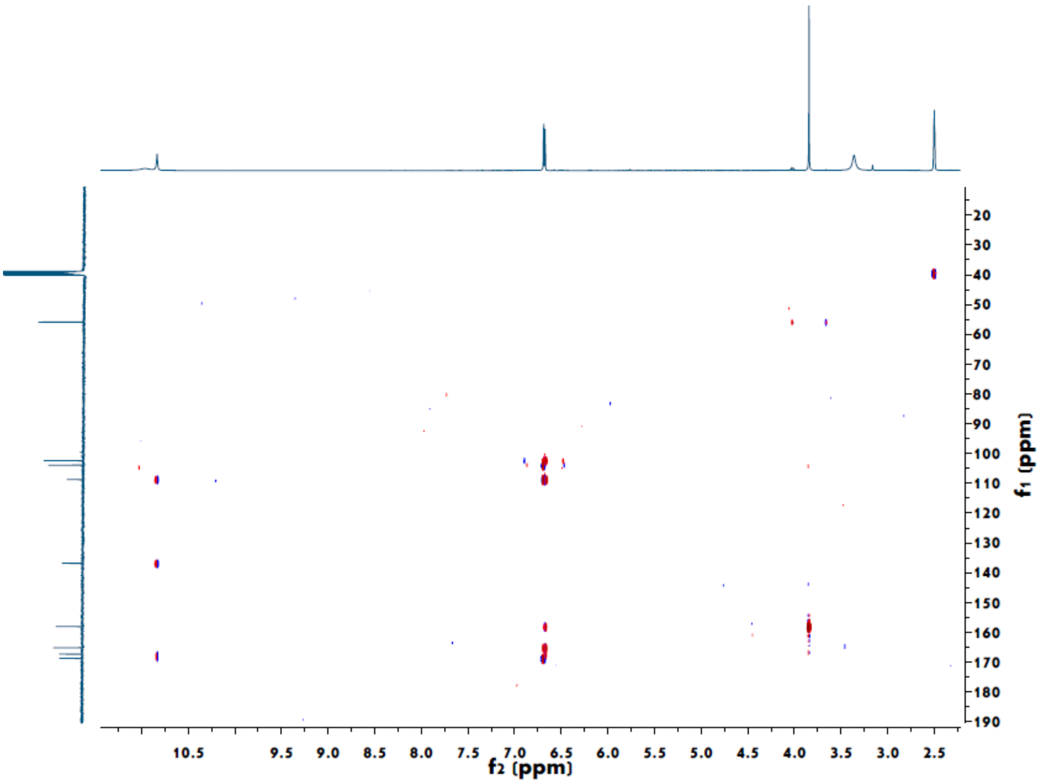

Supplement: File 1 — 1H, 13C, 1H,1H COSY, HSQC and HMBC NMR spectra of the new compounds. [file Beilstein_J_Org_Chem-11-1187-s001.pdf]
